# Structural readiness of CBOs to address health-related social needs in chronic disease management programs

**DOI:** 10.1186/s12913-026-15017-9

**Published:** 2026-06-25

**Authors:** Gianna D’Apolito, Niko Verdecias-Pellum

**Affiliations:** College of Health Solutions, Population Health, Arizona State University, Phoenix, AZ USA

**Keywords:** Community-based organizations, Chronic disease, Health-related social needs, Structural capacity, Organizational capacity, Health service delivery

## Abstract

**Background:**

Addressing health-related social needs (HRSN) such as food insecurity, housing instability, and transportation barriers has become increasingly recognized as essential to improving chronic disease outcomes. Community-based organizations (CBOs) are uniquely positioned to assess and address HRSN due to their embeddedness within local communities. CBOs that provide chronic disease self-management programs may have distinct capacities to integrate HRSN support, tailor interventions to community contexts, and build trusting relationships that enhance client engagement and sustain behavior change. However, limited research has examined the structural and organizational capacities required for CBOs to systematically integrate HRSN-related services in chronic disease management contexts.

**Methods:**

An environmental scan using a structured, purposive search was conducted to identify literature relevant to CBOs, HRSN, and chronic disease across PubMed, Scopus, and targeted Google searches. Of 224 sources identified, 20 were included for analysis. Data were organized using a structured matrix and analyzed through thematic synthesis using a modified Strengths, Weaknesses, Opportunities, and Threats (SWOT) framework to guide coding and distinguish internal and external factors. Findings were subsequently interpreted using the Consolidated Framework for Implementation Research to contextualize results across implementation domains.

**Results:**

CBOs demonstrated variation in capacity to assess and address HRSN. Facilitators included patient navigation services, community-aligned missions, and cross-sector partnerships. Barriers included limited data infrastructure, inconsistent referral processes, and resource constraints. A capacity continuum emerged, illustrating how internal organizational factors and external conditions interact to shape reach and effectiveness. Two key insights included a partnership paradox, in which capacity is often required to form partnerships, and a bidirectional relationship between data capacity and external engagement.

**Conclusions:**

Findings indicate that CBO capacity is shaped by multilevel factors, including organizational systems, policy and funding environments, and the availability of community resources. Strengthening this capacity will require sustained investment in data infrastructure, workforce development, and community-based services, as well as alignment between partnership expectations and organizational readiness. These findings also underscore the importance of addressing structural resource constraints alongside coordination efforts and provide implementation-informed insights to guide future research, policy, and program design aimed at strengthening CBO capacity to deliver HRSN-related services within chronic disease self-management programs.

## Introduction

Chronic diseases affect approximately 60% of the U.S. population and remain a leading cause of morbidity, mortality, increasing healthcare expenditures, and decreased quality of life [1, 2]. Chronic diseases such as diabetes and cardiovascular disease are influenced not only by clinical care, but also by a range of non-medical factors that shape individuals’ capacity to engage in disease prevention and management [3, 4]. In recent years, addressing health-related social needs (HRSN) such as food insecurity, unstable housing, transportation barriers, and utility barriers has become an important strategy in chronic disease management [5, 6]. Recent evidence shows that HRSN are associated with lower medication adherence, greater barriers to care access, and worse utilization outcomes, whereas interventions that improve patient activation and self-management can support sustained behavior change [7–11]. 

HRSN are distinct from broader social determinants of health (SDOH) that represent upstream, structural conditions such as socioeconomic status (SES), education, neighborhood environments, and healthcare access that shape health outcomes at the population level [1]. In contrast, HRSN represent the immediate, individual-level manifestations of these broader conditions [1]. While SDOH influence the distribution of risk and resources across populations, HRSN are actionable within healthcare and community-based service delivery contexts. Addressing HRSN has emerged as a key strategy for mitigating the downstream effects of structural inequities on chronic disease outcomes [1, 5, 6, 11]. 

Unmet HRSN are often interrelated, reflecting immediate needs that arise from broader resource imbalances. Increasing evidence shows that these needs frequently co-occur, compound disease risk, and create cascading effects on health [12, 13]. For example, individuals presenting with a single identified HRSN often have additional unmet needs across domains such as food security, housing, healthcare access, or personal safety [14]. Verdecias et al. (2021) highlight this interconnectivity of HRSN, noting that the presence of one expressed need significantly increases the likelihood of additional unexpressed needs [14]. 

These structural conditions also drive the uneven distribution and clustering of HRSN across populations, contributing to persistent differences in chronic disease outcomes [1, 15–17]. Individuals residing in areas with fewer resources often experience diminished access to stable housing and nutritious foods, which impede chronic disease management and drive adverse outcomes [18–20]. Housing instability can hinder medication adherence and access to essential care, while food insecurity has been linked to poor glycemic control and greater disease severity in diabetes [18–20]. National studies confirm an inverse association between food security and chronic diseases, such as type 2 diabetes mellitus (T2DM), hypertension, and hyperlipidemia, supporting a growing need for comprehensive, community-level interventions [6, 21]. These patterns highlight the need for community-based strategies that address the social conditions shaping chronic disease management.

Community-based organizations (CBOs) operate at the intersection of healthcare and social services and are well-positioned to address HRSN within local contexts. With established relationships in the communities they serve, CBOs can provide accessible, culturally relevant services and build trust with individuals who may face barriers to traditional healthcare systems. These organizations often serve as critical connectors between clinical care and social support systems, helping individuals navigate resources and sustain engagement in care.

CBOs are also key providers of chronic disease self-management (CDSM) programming. Many deliver Chronic Disease Self-Management Education (CDSME) programs, which are designed to support individuals across multiple chronic conditions, as well as diabetes-specific self-management education programs that focus on condition-specific care. In addition to service delivery, CBOs contribute to social cohesion, a protective factor associated with lower incidence of chronic diseases [22]. For example, neighborhoods with higher social cohesion have been found to have a 22% lower incidence of T2DM [23]. These findings underscore the importance of community-based strategies that strengthen social ties and support networks. Given their embeddedness within local communities and capacity for tailored interventions, CBOs are well positioned to support these mechanisms.

While CDMSE programs have demonstrated efficacy [24, 25], their accessibility and adaptability vary widely depending on community resources and infrastructure [26]. These peer-delivered models show promise in community settings, but challenges remain in scaling and sustaining them across diverse contexts. In addition, evolving policy developments and funding mechanisms, including state-level Medicaid initiatives, may create new opportunities for partnership and service delivery, though their availability and sustainability vary across states and may be influenced by changing funding environments [27–29]. However, it remains unclear how CBOs can effectively operationalize these resources to expand their role in chronic disease management [30, 31]. Despite these advances, there remains a limited understanding of the structural and organizational capacity of CBOs to implement HRSN-related services in a systematic and sustained way.

To address this gap, we examined the structural and organizational factors that influence the capacity of CBOs to assess and address HRSN within CDSM programs. We conducted an environmental scan to support an analytic, practice-oriented synthesis of the literature, integrating evidence across diverse contexts. Through thematic analysis, we identified cross-cutting barriers and facilitators and generated conceptual insights related to CBO capacity. Findings were interpreted through an implementation science lens informed by the Consolidated Framework for Implementation Research (CFIR). This approach moves beyond descriptive summary to develop interpretive contributions, including a capacity continuum and insights into partnership dynamics, with implications for implementation and intervention design.

## Methods

### Study design and approach

This study used an environmental scan to identify relevant literature as the primary data source for analysis, rather than as a formal, systematic literature synthesis approach. The environmental scan captured heterogeneous evidence across peer-reviewed and practice-based sources relevant to CBO capacity to address HRSN within CDSM contexts. Rather than aiming for comprehensive coverage and formal appraisal, this approach prioritized the integration of diverse evidence sources to examine patterns across varied organizational and community settings. The environmental scan served as a data identification strategy, while qualitative thematic analysis constituted the primary analytic method.

### Identification and selection of sources

Relevant literature was identified through a structured, purposive search of PubMed, and SCOPUS (covering publications from 2014 to 2024), and supplemented by targeted Google searches (2019–2024) to capture peer-reviewed articles, grey literature and practice-based reports not indexed in bibliographic databases. PubMed and Scopus were selected to provide complementary coverage of biomedical, public health, and interdisciplinary research related to chronic disease management and HRSN, while Google searches supported the inclusion of practice-oriented sources. This approach enabled a focused environmental scan prioritizing relevance to CBO capacity and real-world application, rather than exhaustive database coverage.

Search terms combined keywords related to CBOs, HRSN, and CDSM using Boolean operators (Table 1). Searches were conducted between November 2024 through January 2025, with the final search completed on January 26, 2025. This search process was iterative, with terms refined based on emerging relevance. Table 1 provides representative examples of search strategy.

**Table 1:** Representative search terms used for source identification

Concept Area	Keywords Used
Community-Based Organizations	“community-based organization”, “CBO”, “community organization”
Health-Related Social Needs	“health-related social needs”, “social risk”, “basic needs”, “SDOH”
Chronic disease	“CDSME”, “chronic disease self-management”, “diabetes”, “diabetes management”
Combined searches	Terms from the above categories were combined using Boolean operators (e.g., AND, OR) to identify sources addressing CBOs, HRSN, and chronic disease self-management

References were managed using Zotero (reference management software) and duplicate articles were removed prior to review. Sources were then assessed for relevance to the research question. Eligible studies included those conducted in the United States, focused on adult populations (at least 18 years of age), and described CBOs that delivered or supported chronic disease self-management programs such as Chronic Disease Self-Management Education (CDSME) and Diabetes Self-Management Education (DSME) with relevance to HRSN. Studies that did not address HRSN in the context of CBOs delivering chronic disease management programs were excluded.

Included studies reflected both HRSN activities integrated within CDSM programs and those conducted through broader organizational screening and referral processes. CBOs were defined based on how they were described in the included studies; eligible organizations were those identified as community-based and operating within community settings, rather than clinical-only environments. This approach reflects variation in how CBOs are operationalized in the literature and aligns with the study’s focus on organizations embedded within and serving local communities.

Consistent with the purposive and analytic nature of this environmental scan, this approach was not intended to serve as a formal systematic or scoping review. Accordingly, systematic review reporting guidelines (e.g., PRISMA) were not applied. However, key elements of transparent reporting were incorporated, including documentation of search strategies, selection criteria, and analytic procedures. The study was approved and deemed exempt by the Arizona State University Institutional Review Board.

### Data abstraction and preparation

Relevant information from each source was compiled into a structured matrix to support comparative and thematic analysis. Extracted data included publication year, geographical setting (urban, rural), study setting (clinic-community partnership, community only), study design (quantitative, qualitative, or mixed methods), HRSN screening practices (Yes/No), types of HRSN addressed, presence of chronic disease health-supportive programs (e.g., CDSME, diabetes self-management education) (Yes/No), and organizational processes, resources, and partnerships. The matrix also captured descriptions of barriers and facilitators related to HRSN service delivery.

Two reviewers from the research team independently assessed each source for relevance and extracted data using this matrix. Discrepancies were resolved through regular team meeting discussions and consensus to ensure consistency in interpretation. This process supported structured organization of the data while preserving the heterogeneity of the included sources.

### Analytical approach

Analysis proceeded in a multi-step, sequential process using thematic synthesis with a framework-informed hybrid deductive–inductive approach [32] to coding, followed by framework-based interpretation. First, thematic analysis was conducted on extracted textual data including descriptions of program implementation, organizational characteristics, and reported barriers and facilitators, using a Strengths, Weaknesses, Opportunities, and Threats (SWOT) framework [33] to guide coding. Two reviewers independently coded the data using an iterative approach, applying SWOT-informed categories to distinguish internal organizational factors (strengths and weaknesses) from external contextual influences (opportunities and threats), while allowing for the identification of emergent subthemes within each category. Discrepancies were resolved through regular consensus meetings to ensure consistency in interpretation, and codes were grouped into higher-order themes representing recurring patterns across the literature. To enhance interpretability, SWOT categories were modified and consolidated into facilitators (strengths and opportunities) and barriers (weaknesses and threats).

Finally, themes were mapped to the Consolidated Framework for Implementation Research (CFIR) [34] as a secondary interpretive step. CFIR is a widely used implementation science framework designed to identify multilevel factors that influence the adoption and delivery of interventions [35]. In this study, CFIR domains (i.e., inner setting, outer setting, characteristics of individuals, and implementation process) were used to contextualize findings related to CBO capacity to address HRSN. The inner setting refers to organizational characteristics such as infrastructure, workflows, and resources; the outer setting captures external influences, including policy, funding, and community resource availability; characteristics of individuals reflect the roles, skills, and engagement of staff; and the process domain encompasses activities such as partnership development, coordination, and implementation of services. CFIR did not guide initial coding; rather, it was used to situate emergent themes within a multilevel framework relevant to implementation in community-based settings. Together, this analytical approach enabled the identification of cross-cutting patterns and the development of conceptual insights related to CBO capacity. In this analysis, capacity was not defined as a single measured construct but was derived from patterns reported across the included studies.

## Results

### Characteristics of included sources

From an initial set of identified sources (*n* = 224), a total of 20 were included in the analysis (Table 2 overviews included articles). These sources reflected diverse programmatic and organizational contexts relevant to CBOs addressing HRSN within CDSM programs.

**Table 2:** Articles meeting inclusion in the analysis

Lead Author (Year)	Title [Manuscript Reference #]	Inclusion Description
Jessica M Sautter (2024)	Health outcomes reported by healthcare providers and clients of a community-based medically tailored meal program [46]	One HRSN (food insecurity) addressed
Allison Slater (2022)	Latino Health Access: Comparative Effectiveness of a Community-Initiated Promotor/a-Led Diabetes Self-management Education Program [47]	Self-management education focused, which assessed HRSN
Dea Papajorgji-Taylor (2021)	Bridge to Health/ Puente a la Salud: Rationale and design of a pilot feasibility randomized trial to address diabetes self-management and unmet basic needs among racial/ethnic minority and low-income patients [31]	Multiple HRSN addressed; Navigation services; Diabetes self-management support
Linda Zayed (2020)	Health Assessment of the Arab American Community in Southwest Chicago [49]	Assessed HRSN (i.e. affordable housing)
Kate Dupont Phillips (2022)	Healthy Communities Delaware: Accelerating Place-Based Efforts to Improve the Vital Conditions for Health, Well-Being and Equity [43]	Vital Conditions for Health and Well-Being (transportation, learning, basic needs for health and safety, housing, belonging)
Shayla N M Durfey (2021)	Health Care and Community-Based Organization Partnerships to Address Social Needs: Medicare Advantage Plan Representatives’ Perspectives [45]	Challenges of health entities, CBO partnerships, and Medicare Advantage
Deborah Onakomaiya (2024)	Stakeholder Perspectives on the Impact of COVID-19 on the Implementation of a Community-Clinic Linkage Model in New York City [38]	HRSN included; community-focus
Loren Saulsberry (2022)	“Everything in One Place”: Stakeholder Perceptions of Integrated Medical and Social Care for Diabetes Patients in Western Maryland [41]	Multiple HRSN addressed
Waseem Sous (2021)	Integrated Care Management to Improve Diabetes Outcomes in Refugee and Immigrant Patients (I-Care) [54]	Community-focus; Navigation services
Kim Hanh Nguyen (2021)	The role of community-based organizations in improving chronic care for safety-net populations [26]	Multiple HRSN addressed; Navigation services
Kameswari A. Potharaju (2022)	Assessing Alignment of Patient and Clinician Perspectives on Community Health Resources for Chronic Disease Management [36]	Multiple HRSN addressed; CBO partnership
Valerie L Polletta (2021)	Facilitators and Barriers of a Chronic Care Management Intervention Addressing Diabetes Among Mexican-Origin Adults [55]	Community-focus; CBO partnership
Aisha Bhimla (2017)	Addressing the Health Needs of High-Risk Filipino Americans in the Greater Philadelphia Region [40]	Community-focus; HRSN assessment
Kristin Pullyblank (2022)	Implementation of Evidence-Based Disease Self-Management Programs in a Rural Region: Leveraging and Linking Community and Health Care System Assets [44]	Multiple HRSN addressed; CBO partnership
Eric E. Calloway (2024)	Benefits of using both the Hunger Vital Sign and brief nutrition security screener in health-related social needs screening [50]	One HRSN (food insecurity) assessed
Jessica Sattler (2023)	A Nebraska Community Proactively Addresses Social Drivers of Health [56]	Multiple HRSN addressed; CBO partnership
Katie Huber (2024)	Addressing Housing-Related Social Needs Through Medicaid: Lessons From North Carolina’s Healthy Opportunities Pilots Program [39]	One HRSN (housing) addressed
Courtney Lyles (2024)	Building a Client Resource and Communication Platform for Community-Based Organizations to Address Health and Social Needs: Co-Design Study [48]	Digital health focus; creating CBO resources
Elham Hatef (2024)	Piloting a Clinical Decision Support Tool to Identify Patients With Social Needs and Provide Navigation Services and Referral to Community-Based Organizations: Protocol for a Randomized Controlled Trial [37]	Protocol; Multiple HRSN assessed; CBO partnership
Justin Dower (2022)	Patient Voices: Doctors and Diabetes Management [42]	Qualitative perspectives on Diabetes care and management; HRSN assessment

Most sources (*n* = 14; 70%) were published between 2022 and 2024, indicating increasing attention to this area in recent years. Diabetes was the most frequently represented condition (60%, *n* = 12), reflecting both its prominence in community-based self-management programming and its inclusion in the search strategy. Searches included CDSM and diabetes-specific terms, as diabetes is commonly represented within CDSM programming. The search strategy also incorporated broader CDSM terms to capture relevant CBO-based programs beyond diabetes alone. The majority of studies were conducted in urban settings (*n* = 16; 80%), with a smaller number including both urban and rural populations. Study designs varied and included program evaluations, needs assessments, and implementation-focused research.

Across the included sources, many described the implementation of specific programs aimed at screening for or addressing HRSN, or examined the prevalence of HRSN within defined populations. A small number of studies (*n* = 3, 15%) focused on a single HRSN which was most commonly food insecurity. While the majority (*n* = 17, 85%) addressed multiple, co-occurring needs. For example, one study implemented the Hunger Vital Sign and Brief Nutrition Security Screener across multiple CBOs to identify food-related needs, while another described the “Living Well” initiative, which coordinated services such as nutrition, physical activity, medication management, and social support through a network of CBOs. Some programs were tailored to specific populations, including initiatives focused on immigrant and refugee communities.

These sources provided the basis for identifying cross-cutting patterns related to organizational capacity, including recurring barriers and facilitators influencing the ability of CBOs to address HRSN. The inclusion of diverse CBO types and resource contexts was intentional to capture the range of organizational capacity observed in practice. Themes derived from our analysis are presented below and are organized to distinguish internal organizational factors from external contextual influences.

### Populations served

Across included sources, characteristics of the populations served were described as shaping the context in which HRSN were identified and addressed. These characteristics influenced both the complexity of needs expressed and the availability of resources to respond.

Several sources described programs serving populations with a high prevalence of unmet HRSN, including food insecurity, housing instability, and limited access to healthcare. In these contexts, CBOs were often required to address multiple, co-occurring needs, increasing demands on organizational capacity. At the same time, the availability of community resources was often limited, constraining the ability to respond to identified needs.

In contrast, some sources described settings with more robust community resource networks, which supported more effective referral and coordination of services. Together, these findings reflect how population characteristics were associated with both service demand and the availability of community resources.

### Modified SWOT outcomes

**Fig. 1:**
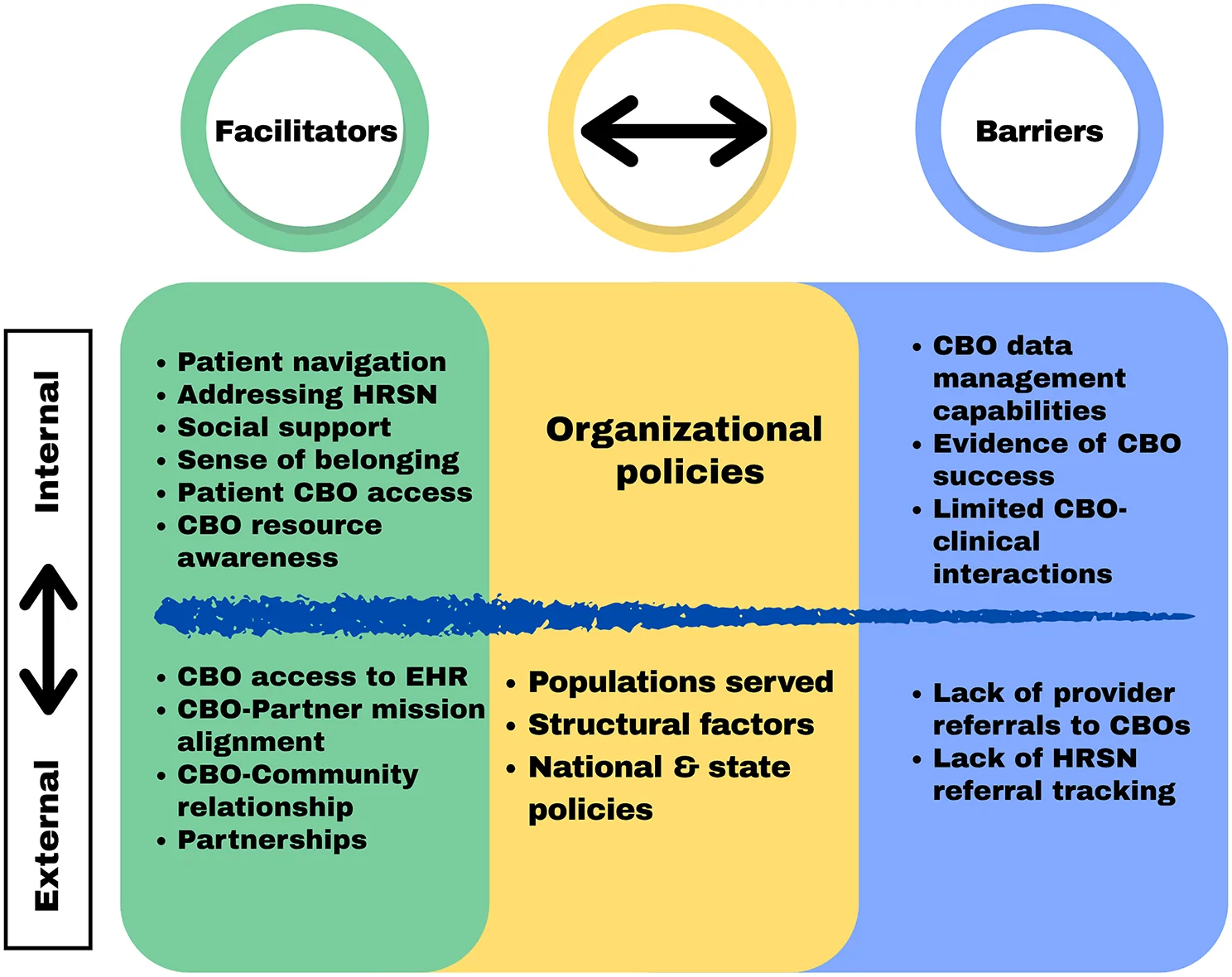
Factors influencing health-related social needs service implementation in CBOs. Abbreviations: HRSN = Health-Related Social Needs; CBO = Community-based Organization; EHR = Electronic Health Records

**Fig. 2:**
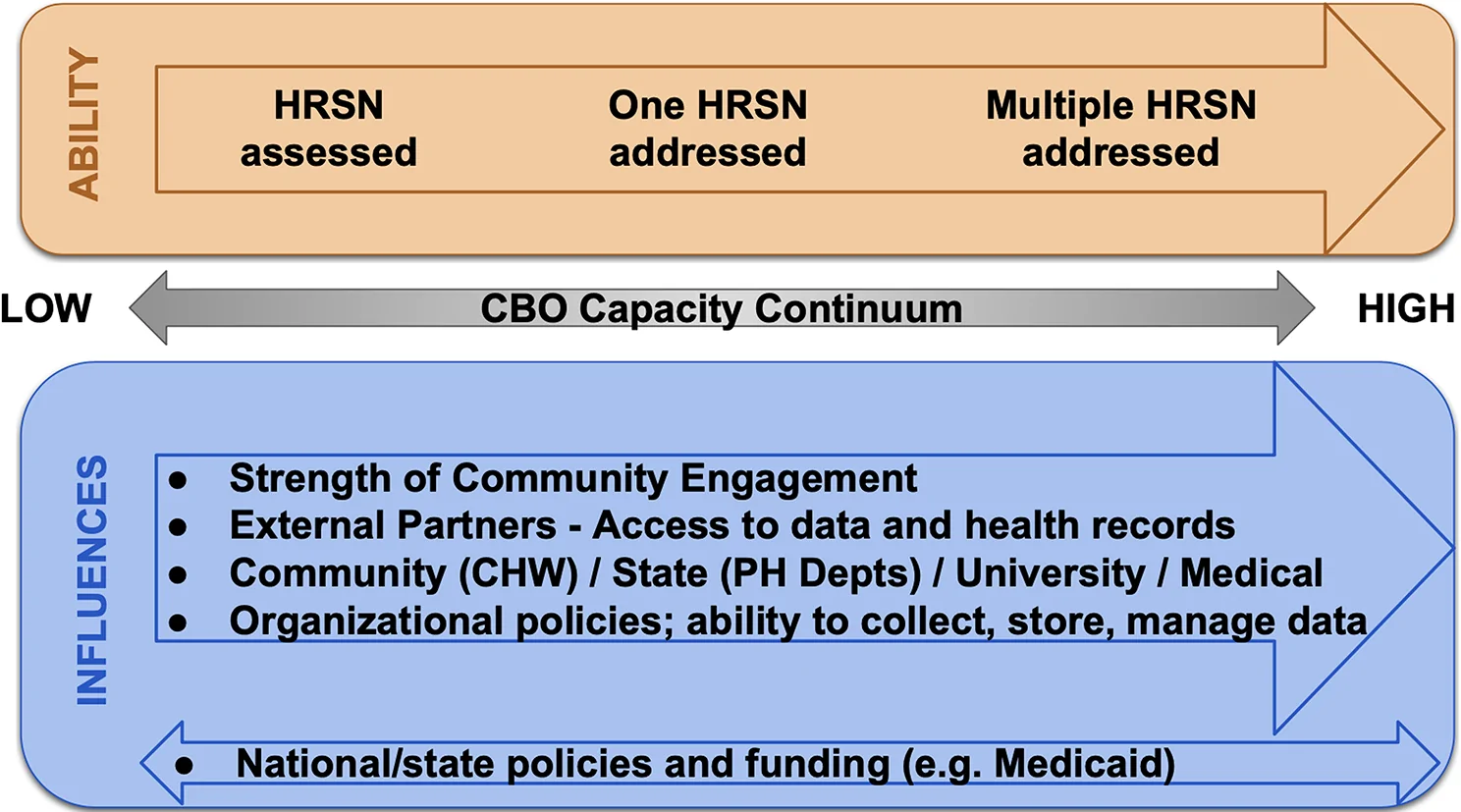
CBO capacity continuum. Abbreviations: (HRSN= Health-Related Social Needs; CHW= Community Health Worker; PH=Public Health)

Figure 1 presents the results of the modified SWOT analysis. Findings were organized into facilitators and barriers to implementation, reflecting internal (organizational), external (contextual), and other influences that operate across levels. For example, barriers such as “limited CBO–clinical interactions” may be shaped by both internal outreach capacity and external partner engagement. Populations served, structural factors, and policy environments, were identified as key influences that can either strengthen or constrain implementation depending on context. This is described further in the discussion of the CBO capacity continuum (Fig. 2).

Across sources, several organizational factors, including funding for staff and training, understanding of community needs, and potential for strategic partnerships, were commonly reported as facilitators. In contrast, barriers were most often associated with limitations in data management, variability in referral processes, and broader structural constraints. These facilitators and barriers are described below.

#### Facilitators

Facilitators included internal strengths (i.e., positive attributes enhancing CBO capacity to address community needs) and external opportunities that supported CBO capacity to address HRSN. At the organizational level, internal strengths revealed that patient navigation services, including community health workers and promotoras, were consistently identified as key facilitators, supporting individuals in navigating complex health and social systems [31]. CBO capacity to assess and address HRSN varied across organizations, with structured screening and response processes associated with greater reach and effectiveness [36–40]. The ability to create safe, accessible environments that foster social support and belonging were also reported as contributing to improved engagement [41–43]. Other organizational facilitators included resource awareness and data capacity. Knowledge of available community resources supported effective referrals, while access to client data and health records enabled more coordinated and responsive care, even without formal partnerships [31, 44–46]. 

At the contextual level, external opportunities revealed alignment between CBO and partner organizational missions supported collaboration, while strong understanding of local community needs facilitated outreach and engagement [26, 39, 43]. Cross-sector partnerships, including collaborations with healthcare systems, academic institutions, and other CBOs, were consistently identified as key facilitators of service delivery and capacity-building [46, 47]. 

#### Barriers

Barriers included internal limitations and external constraints that hindered CBO capacity to address HRSN. Internally, at the organizational level, insufficient data infrastructure was a major barrier, including challenges related to data collection, storage, and interoperability [38, 44, 48]. Limited staffing, training, and standardized workflows also constrained implementation. In addition, external partners often required evidence of organizational effectiveness prior to collaboration, creating challenges for lower-capacity organizations seeking to build partnerships [45]. 

At the contextual level (externally), variability in referral practices across healthcare providers was a recurring barrier. Informal referral pathways, reliance on word-of-mouth knowledge, and inconsistent documentation and follow-up procedures limited coordination between healthcare organizations and CBOs [36, 44]. 

At the most distal level, broader structural conditions, including housing policies, socioeconomic inequities, and intergenerational poverty, constrained both resource availability and the ability of CBOs to respond to identified HRSN [26, 43, 49]. 

#### Alignment with CFIR: an interpretive lens

When mapped to CFIR domains, several key themes emerged that reflect multilevel determinants of CBO capacity to address HRSN, consistent with the internal, external, and cross-cutting structure illustrated in Fig. 1. Table 3 summarizes this alignment by mapping SWOT-informed facilitators and barriers to CFIR domains. This mapping illustrates how organizational factors, external conditions, and implementation processes interact to shape variation in CBO capacity.

**Table 3:** Findings mapped to modified SWOT categories and CFIR domains

CFIR Domain	Brief Definition	Modified SWOT Categories	
		Facilitators (Strengths/Opportunities)	Barriers (Weaknesses/Threats)
Intervention Characteristics	Features of programs or interventions that influence implementation (e.g., adaptability, complexity)	Adaptability of CDSME and HRSN-integrated programs; culturally tailored interventions; peer-delivered program models	Complexity of integrating HRSN screening and response into existing programs; variability in program design and implementation
Inner Setting	Organizational characteristics including infrastructure, workflows, and resources	Patient navigation services (e.g., CHWs, promotoras); internal capacity to assess and address HRSN; structured screening and referral workflows; flexible organizational policies; knowledge of community resources	Limited data infrastructure (data collection, storage, interoperability); insufficient staffing and training; lack of standardized workflows; rigid or outdated organizational policies
Outer Setting	External environment including policy, funding, and community resources	Medicaid-related funding mechanisms; policy support for HRSN integration; strong community resource networks; alignment between CBO and partner missions; access to community resources	Limited availability of community resources (e.g., housing, food access); funding instability; structural inequities; misalignment between healthcare and community systems
Characteristics of Individuals	Skills, roles, and beliefs of individuals delivering services	Community health workers, promotoras, and navigators facilitating trust and engagement; culturally competent service delivery; strong community relationships	Limited workforce training; staff capacity constraints; variability in staff ability to address complex HRSN needs
Process	Implementation activities such as partnerships, coordination, and engagement	Cross-sector partnerships (healthcare, public health, academic, CBOs); referral coordination; stakeholder engagement; use of data to support care coordination	Inconsistent referral processes; reliance on informal referral pathways; lack of follow-up and feedback loops; partnership barriers requiring prior capacity (partnership paradox)
Cross-Cutting Factors *(spanning domains)*	Factors influencing multiple domains simultaneously	Data sharing and access to patient information; resource awareness; community engagement and trust; integration of clinical and social data systems	Data-sharing burden on CBOs; lack of interoperability; increased workload from referrals; mismatch between referral volume and organizational capacity; population-level resource constraints

Internal organizational factors, including data infrastructure, internal workflows, workforce capacity, and patient navigation services, aligned with the inner setting domain. These factors relate to constructs such as readiness for implementation, available resources, and implementation climate. Variation in these elements across organizations was observed with differences in structural capacity and operational readiness.

External contextual factors, including funding mechanisms, policy environments, availability of community resources, and structural inequities, corresponded to the outer setting domain. These findings were consistent with external policy and incentives, client needs and resources.

Cross-cutting factors, including data sharing, resource awareness, and community engagement, operated across both internal and external domains. These elements reflect interactions across inner setting, outer setting, and process domains, particularly in relation to coordination, communication, and engagement across sectors.

The role of community health workers, promotoras, and other client-facing staff aligned with the characteristics of individuals domain, highlighting workforce skills, knowledge, and trust-building in supporting service delivery.

Cross-sector partnerships and referral coordination aligned with the process domain, particularly in relation to stakeholder engagement and execution of implementation strategies. The partnership characteristics identified in this analysis highlight connections between process-related dynamics, internal capacity for referral coordination, and external system expectations for established capacity.

Together, these findings capture the interconnected nature of multilevel influences on CBO capacity and support a CFIR-informed understanding of implementation in community-based settings.

#### CBO capacity continuum

Our analysis revealed notable variation in the capacity of CBOs, with higher-capacity organizations demonstrating greater ability to assess and address HRSN. Factors influencing CBO capacity were synthesized into a continuum (Fig. 2) that illustrates how variation in organizational capacity relates to the reach and effectiveness of CBOs in addressing HRSN. As depicted in Fig. 2, progression along the continuum reflects increasing ability to move from assessing HRSN to addressing one or multiple needs. This continuum reflects variations identified in internal organizational factors, external contextual influences, and other cross-level factors, consistent with CFIR domains.

A key finding was the link between organizational capacity and the level of community engagement, particularly the extent to which CBOs involved the populations they served in program planning and delivery. This construct was not directly measured but derived from qualitative descriptions in the included studies, reflecting how CBOs engaged community members in program delivery, outreach, and service design. In addition, the breadth and quality of external partnerships, including collaborations with community health workers, public health departments, academic institutions, medical systems, clinics, and peer CBOs, emerged as central to capacity building. These constructs were not directly measured but were derived from qualitative descriptions in the included studies, with breadth reflecting the range of partner types and quality reflecting reported characteristics such as coordination, alignment of goals, and strength of collaboration. These factors align with both the outer setting and process domains, reflecting the role of external networks and stakeholder engagement [39, 42, 50]. 

Data access and health record integration represented a distinct, though related, driver of capacity. Organizations with advanced data systems and the ability to integrate clinical and non-clinical data demonstrated higher operational effectiveness and engagement with external partners [44, 48]. This finding aligns with the inner setting domain, particularly constructs related to available resources and organizational infrastructure, while also reflecting coordination processes across organizations.

Notably, a paradox emerged in which external partners, particularly in healthcare and academic settings, tended to favor collaborations with CBOs that already exhibited established internal capacity, particularly in data infrastructure [45]. This partnership paradox reflects the interaction between outer setting expectations and inner setting capacity, where requirements for partnership may inadvertently constrain opportunities for lower-capacity organizations.

Internal organizational policies also played a key role, particularly those related to data collection, storage, and use. These policies demonstrated a bidirectional influence: when designed with flexibility and aligned with community needs, they enhanced organizational capacity, whereas rigid or outdated policies constrained growth and adaptability. These findings further align with the inner setting domain.

Finally, state and national policy environments, especially those related to funding mechanisms such as Medicaid expansion, substantially shaped CBO capacity. Polices that enabled increased resource flow to community organizations, such as through Medicaid waivers or state-level contracts, positively influenced infrastructure and service reach [39, 41, 43]. However, given the potential fluctuations of such policies, their influence was modeled as bidirectional within the capacity continuum to reflect their dynamic impact on both high- and emerging or limited-capacity CBOs. These findings correspond to the outer setting domain.

## Discussion

This study advances an analytical understanding of CBO capacity to address HRSN within chronic disease management by identifying a fundamental misalignment between how CBOs are conceptualized within health systems and the scope of their actual service delivery roles. Rather than functioning solely as community assets, CBOs operate as integral service delivery partners whose effectiveness depends on infrastructure, referral systems, and operational workflows. These findings suggest that CBO capacity is not simply an organizational characteristic, but is shaped by how health systems define, engage, and resource these organizations, as well as how CBOs are structured to deliver HRSN-related services within CDSM programs. As a result, efforts to support CBO delivery of HRSN services must move beyond recognizing CBOs as partners to aligning system expectations with the structural realities that shape their capacity.

Organizational capacity, particularly in data infrastructure, partnership readiness, and workforce development, emerged as a central determinant of implementation effectiveness. However, these findings further indicate that capacity is not solely internally determined but is dynamically shaped through interactions with external policy, funding, and partnership environments. This builds on prior research emphasizing the importance of integrating structural and social interventions in chronic disease prevention and management, including evidence demonstrating how social and environmental conditions influence disease risk and control and how healthcare–CBO partnerships support chronic care delivery [6, 26]. Hill-Briggs et al. (2020) highlight the role of socioeconomic and environmental conditions in shaping chronic disease outcomes [6]. Similarly, Nguyen et al. (2021) underscore the importance of addressing HRSN and fostering healthcare–CBO partnerships in effective chronic care management [26]. Extending this work, our findings suggest that implementation effectiveness is not simply a function of organizational readiness, but of the degree to which internal capacity and external system-level conditions are aligned and mutually reinforcing.

Barriers such as limited data infrastructure and inconsistent workflows point to challenges in organizational readiness, while external constraints, including policy variability, funding instability, and partnership requirements, reflect broader structural influences on CBO operations. Taken together, these findings challenge the conceptualization of CBO capacity as an internal organizational attribute, instead suggesting that capacity is relational and co-produced through interactions between organizational infrastructure and system-level conditions. In this context, policy, funding, and resource availability do not simply influence implementation, but actively shape the boundaries of what is feasible for CBOs to deliver. These findings suggest that expectations for CBO capacity should be context-dependent, reflecting variation in organizational resources, infrastructure, and community environments.

This analysis contributes two key insights that expand existing literature on CBO capacity. First, the partnership paradox identified in this study highlights a reinforcing interaction between inner and outer setting factors. External partners, particularly healthcare and academic organizations, often require evidence of strong internal capacity before forming collaborations. This dynamic may limit opportunities for resource-constrained CBOs to engage in partnerships that could otherwise support capacity-building, reinforcing a cycle in which organizations with fewer resources are systematically excluded from opportunities for growth and integration into care delivery systems. This finding suggests that partnership structures themselves may function as mechanisms that reproduce inequities, rather than neutral pathways for integration. More broadly, this pattern suggests that CBO capacity is not solely an organizational attribute but is structurally distributed across systems and communities. Partnership models that rely on existing capacity as a prerequisite for engagement may inadvertently reproduce inequities by privileging well-resourced organizations while limiting opportunities for those serving higher-need populations. As a result, CBOs operating in under-resourced settings may face compounded barriers to both service delivery and capacity-building. These observations are presented as an interpretive implication rather than a prescriptive or tested model, suggesting that partnership structures could support incremental engagement aligned with organizational capacity.

Second, the findings illustrate the bidirectional relationship between data capacity and external engagement. Robust data systems were frequently described as prerequisites for partnerships, while in other contexts, they also developed as a result of collaboration with external partners. At the same time, evidence from the Accountable Health Communities (AHC) Model suggests that participation in data-sharing initiatives and increased referral volume can introduce additional workload, particularly for smaller organizations with limited staffing and infrastructure [51, 52]. Together, these findings indicate that data systems and referral processes function as both enablers and constraints of capacity, revealing a feedback loop in which data infrastructure both facilitates and is shaped by external engagement. This dynamic underscores that expectations for data sharing are not neutral requirements, but system-level demands that can either enable or constrain participation depending on organizational resources.

Building on this, these findings also raise important considerations for how CBO capacity is measured and evaluated. Common indicators such as data infrastructure, referral volume, and reporting capabilities may disproportionately reflect the capacities of well-resourced organizations, potentially obscuring the contributions and constraints of smaller or resource-constrained CBOs. Without accounting for variation in organizational context, these metrics may inadvertently reinforce existing inequities by privileging organizations that are already positioned to meet formalized reporting and data-sharing expectations. This suggests that prevailing approaches to measuring capacity may obscure structural disadvantage, highlighting the need for evaluation frameworks that account for context rather than assuming uniform organizational readiness. These findings should also be interpreted in light of the study’s methodological approach. As an environmental scan, this analysis prioritized the identification and synthesis of diverse peer-reviewed and practice-based sources rather than conducting a formal evidence synthesis with quality appraisal. Consequently, the findings are intended to generate practice-informed insights and conceptual understanding rather than assess the strength of evidence supporting individual conclusions.

Patient navigation services emerged as a central component of CBO capacity. Roles such as community health workers and promotoras were consistently described as facilitating engagement, coordinating referrals, and linking individuals to services. Evidence from the AHC Model further supports the importance of navigation while highlighting that its effectiveness depends on the availability of community resources [51, 52]. In resource-constrained settings, navigation alone may be insufficient to address identified needs. These findings support reframing patient navigation from a supplemental role to a core structural component of service delivery. However, their effectiveness is contingent on the availability of underlying community resources, indicating that navigation alone cannot compensate for structural service gaps. This reinforces the need for parallel investment in community infrastructure to ensure that identified needs can be meaningfully addressed.

External policy and funding environments also play a critical role in shaping capacity. A subset of studies indicated that Medicaid-related funding mechanisms may support investment in CBO infrastructure and service delivery. For example, North Carolina’s Healthy Opportunities Pilot [53] demonstrates how reimbursement models and data-sharing networks can strengthen partnerships and expand service capacity. However, the evolving nature of such initiatives, including changes or pauses in funding, highlights the instability of these mechanisms and the importance of sustained investment in community-based infrastructure.

These findings also highlight that CBO capacity is not only shaped by funding and coordination mechanisms, but by the availability and accessibility of community resources themselves. In some settings, CBOs and healthcare organizations may be aware of client needs but lack sufficient services to address them, indicating that structural resource gaps, rather than coordination alone, constrain implementation.

From an implementation perspective, strategies to strengthen CBO capacity to deliver HRSN services within CDSM programs should be tailored across CFIR domains. Investments in data systems and workforce development target the inner setting, while policy and funding mechanisms address outer setting constraints. Structured partnership models and intermediary organizations may support the process domain by facilitating engagement between healthcare systems and emerging CBOs. Importantly, these findings indicate that coordination alone is insufficient to address unmet HRSN when underlying community resources are limited or inaccessible. This challenges prevailing implementation models that prioritize coordination without addressing structural service gaps.

These findings highlight the need to reconsider how implementation frameworks such as CFIR account for resource scarcity. While CFIR captures multilevel influences on implementation, resource availability is often conceptualized as a contextual factor rather than a binding constraint. In resource-limited settings, however, the absence of services may fundamentally limit the feasibility of implementation within CBO-delivered chronic disease self-management programs, regardless of coordination or organizational readiness. This identifies a critical gap in prevailing implementation frameworks, which may insufficiently account for contexts in which structural resource scarcity constrains the ability to act on identified needs. CBOs are essential for delivering HRSN-related services within chronic disease self-management programs, and their contributions warrant equitable resourcing and recognition. Healthcare systems, in turn, should calibrate expectations to the capacities and expertise of individual CBOs, supporting their ability to deliver services effectively within community-based settings.

Several limitations should be considered when interpreting the findings of this analysis. First, generalizability may be limited by the heterogeneity of CBOs and by variation in geographic and policy contexts and resources. To address this, we developed a capacity continuum that explicitly accounts for such contextual variation and allows for relative comparisons across settings. Second, this study does not allow for causal inference, and findings reflect patterns reported in the literature rather than direct observation. Additionally, because this study employed an environmental scan rather a systemic or scoping review, no formal assessment of study quality was conducted. As a result, included sources may vary in methodological rigor, and findings should be interpreted as an analytic synthesis of the available literature rather than a weighted assessment based on evidence quality. While the strategy used serves as an important initial step, future research should include in-depth qualitative methods, such as stakeholder interviews or case studies, to explore the factors influencing CBO capacity and implementation effectiveness in greater depth and nuance. Third, key factors such as organizational age and funding levels were not consistently reported and could not be systematically examined. Fourth, the search was limited to PubMed and Scopus, which may have excluded relevant studies indexed in other databases (e.g., CINAHL, PsycINFO), although this was partially mitigated through targeted Google searches and reference list review. Lastly, the policy, funding, and programmatic landscapes that shape CBO operations are subject to change over time. As such, the findings presented here represent a snapshot of a dynamic landscape. Ongoing assessments will be necessary to capture evolving trends. By synthesizing evidence from the past decade, this study provides a foundation for understanding historical and emerging patterns that can inform future research and implementation efforts.

Overall, this study advances understanding of how structural and organizational factors shape CBO capacity to address HRSN within CDSM contexts by demonstrating that capacity is not static or organization-bound, but dynamically produced through interactions between internal infrastructure and external system conditions. By identifying feedback loops, structural constraints, and relational dynamics, these findings offer a more nuanced conceptualization of capacity that informs how health systems engage, evaluate, and resource CBOs in efforts to support the delivery of HRSN services. Integrating evidence across diverse sources, this study further advances a multilevel, implementation-informed perspective that highlights key determinants of capacity and informs future research, policy, and program design.

## Conclusion

CBOs play a critical role in supporting chronic disease self-management through their ability to address HRSN alongside programs such as CDSME and DSME. This analysis demonstrates that CBO capacity varies across settings and is shaped by the interaction of organizational infrastructure, external partnerships, and broader resource environments. By identifying a capacity continuum, this study highlights how differences in readiness and capability influence the extent to which CBOs can move from assessing to addressing client needs within chronic disease management contexts.

These findings underscore the need for sustained investment in data systems, workforce development, and community infrastructure, as well as alignment between program demands and organizational capacity. Strengthening CBO capacity to address HRSN within CDSM programs will require not only coordination across sectors, but also intentional strategies to address structural resource constraints and support the delivery of integrated, community-based services. Continued research and cross-sector collaboration will be essential to advance implementation strategies and enhance the effectiveness and sustainability of CBO-led chronic disease management efforts.

## Data Availability

All data generated and analyzed during this study are included in this published article.

## Abbreviations

HRSN: Health-related social needs
CBO(s): Community-based organization(s)
CDSME: Chronic Disease Self-Management Education
CDSM: Chronic Disease Self-Management
SWOT: Strengths, Weaknesses, Opportunities, and Threats
CFIR: The Consolidated Framework for Implementation Research
SES: Socioeconomic status
T2DM: Type 2 Diabetes Mellitus
